# Protective effect of bevacizumab on chemotherapy-related acute exacerbation of interstitial lung disease in patients with advanced non-squamous non-small cell lung cancer

**DOI:** 10.1186/s12890-019-0838-2

**Published:** 2019-04-02

**Authors:** Shohei Hamada, Hidenori Ichiyasu, Tokunori Ikeda, Megumi Inaba, Kosuke Kashiwabara, Tomoki Sadamatsu, Nahoko Sato, Kimitaka Akaike, Hiroko Okabayashi, Koichi Saruwatari, Yusuke Tomita, Sho Saeki, Naomi Hirata, Takeshi Yoshinaga, Kazuhiko Fujii

**Affiliations:** 1 Department of Respiratory Medicine, Kumamoto University Hospital, Faculty of Life Sciences, Kumamoto University, 1-1-1, Honjo, Chuo-ku, Kumamoto, 860-8556 Japan; 2 Department of Clinical Investigation (Biostatistics), Kumamoto University Hospital, Kumamoto, Japan; 3 Division of Respiratory Medicine, Kumamoto Chuo Hospital, Kumamoto, Japan; 4 Department of Respiratory Medicine, Kumamoto Regional Medical Center, Kumamoto, Japan; 5 Department of Respiratory Medicine, Minamata City General Hospital and Medical Center, Minamata, Japan

**Keywords:** Non-small cell lung cancer, Interstitial lung disease, Acute exacerbation, Bevacizumab, Vascular endothelial growth factor

## Abstract

**Background:**

Acute exacerbation of interstitial lung disease (AE-ILD) is the most serious complication in lung cancer patients with pre-existing ILD receiving chemotherapy. The role of vascular endothelial growth factor (VEGF) in pathogenesis of AE-ILD is conflicting. The influence of bevacizumab (Bev), a monoclonal antibody against VEGF, on lung cancer patients with pre-existing ILD remains unclear. We examined the effect of Bev on reducing AE-ILD risk in non-squamous non-small cell lung cancer (NSCLC) patients receiving chemotherapy.

**Methods:**

We analysed incidence of AE-ILD and outcomes of 48 patients with advanced non-squamous NSCLC with ILD who received first-line chemotherapy with (Bev group, *n* = 17) and without (non-Bev group, *n* = 31) Bev between July 2011 and July 2016. Gray’s test, which was competing risk analysis during the study period, was performed for both groups.

**Results:**

The most common regimen used for first-line chemotherapy was the combination of carboplatin plus pemetrexed (PEM) in both groups. The incidences of chemotherapy-related AE-ILD 120 days after first-line chemotherapy initiation were significantly lower in the Bev than in the non-Bev groups (0% vs. 22.6%, *p* = 0.037, Gray’s test). However, there were no differences in development of progressive disease of lung cancer and other events as the competing risk factors of AE-ILD between the two groups. Only patients receiving PEM-containing regimens also showed a significant difference in the incidence of AE-ILD between the two groups (*p* = 0.044). The overall-cumulative incidence of AE-ILD during the first-line and subsequent chemotherapy was 29.2% (14 of the 48). The median progression-free survival was significantly longer in the Bev than in the non-Bev groups (8.0 vs. 4.3 months, *p* = 0.026).

**Conclusions:**

The addition of Bev to chemotherapy regimens may reduce the risk of chemotherapy-related AE-ILD in patients with lung cancer.

**Electronic supplementary material:**

The online version of this article (10.1186/s12890-019-0838-2) contains supplementary material, which is available to authorized users.

## Background

Interstitial lung disease (ILD), particularly idiopathic pulmonary fibrosis (IPF), is a frequent comorbidity in patients with lung cancer [1, 2]. It poses a major impediment to the treatment of lung cancer, because chemotherapy-related acute exacerbation of ILD (AE-ILD) occurs in 5.6–43% of patients and leads to death in 27.9% [3–5]. Kudoh et al. [3] also reported that pre-existing ILD is a strong risk factor for AE-ILD in patients with non-small cell lung cancer (NSCLC; odds ratio, 4.80–25.27) compared with those without ILD. In clinical practice, lung cancer patients with pre-existing ILD have been treated carefully with cytotoxic chemotherapy. However, there is no established standard regimen for patients with lung cancer with ILD in view of the risk of AE.

The mechanism of AE-ILD including IPF remains to be elucidated. Previous studies in AE-IPF have demonstrated that several pathophysiologies, including epithelial damage and immunogenic dysregulation, were activated [6]. Recently, vascular endothelial growth factor (VEGF) has been thought to have an important role in pathogenesis of AE-IPF [7]. In the lung, VEGF is produced mainly in epithelial cells, whereas endothelial cells are considered its major target. Expression of VEGF is associated with angiogenesis and positive remodelling of damaged tissues; however, it also increases vascular permeability and pulmonary oedema, resulting in acute lung injury [8, 9]. Increasing levels of plasma VEGF in acute respiratory distress syndrome (ARDS) are associated with a worse outcome [10], and in animal models, inhibition of VEGF using a soluble receptor to VEGF reduces bleomycin-induced lung injury and fibrosis [11]. In contrast, other studies from animal models as well as patients with ARDS have shown that decreased levels of VEGF in the lung, which are attributed to diffuse alveolar damage, are related rather to a worse outcome [12, 13]. Therefore, VEGF is assumed to have dual roles in lung injury and fibrosis. Furthermore, Barratt et al. [14] reported that an imbalance of VEGF splice isoforms is critical for development of pulmonary fibrosis. However, the influence of inhibiting function of VEGF on AE-ILD has not been fully examined.

We focused on patients with advanced non-squamous NSCLC with pre-existing ILD who received bevacizumab (Bev), a monoclonal antibody targeting VEGF. We hypothesised that chemotherapy regimens containing Bev might reduce the incidence of AE-ILD by inhibiting function of VEGF. We conducted a multicentre retrospective study to validate this hypothesis and whether Bev-containing regimens can constitute novel candidate chemotherapy in patients with non-squamous NSCLC and ILD.

## Methods

### Patients

**Fig. 1:**
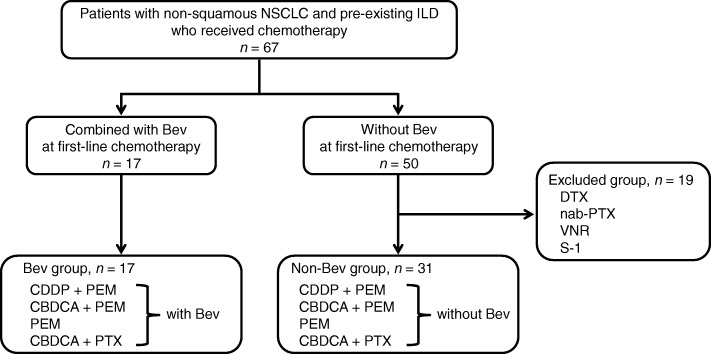
Inclusion and exclusion flow diagram. NSCLC, non-small cell cancer; ILD, interstitial lung disease; Bev, bevacizumab; CDDP, cisplatin; PEM, pemetrexed; CBDCA, carboplatin; PTX, paclitaxel; DTX, docetaxel; VNR, vinorelbine

We reviewed retrospectively medical records of patients with non-squamous NSCLC and pre-existing ILD who received chemotherapy at Kumamoto University Hospital and the three affiliated hospitals: Kumamoto Chuo Hospital, Kumamoto Regional Medical Center, Minamata City General Hospital and Medical Center, between July 2011 and July 2016. The ethics review boards approved the access of medical records. The study subjects were registered consecutively according to the following inclusion criteria: histological or cytological confirmation of advanced non-squamous NSCLC, diagnosis of ILD, no prior chemotherapy, age ≤ 75 years, Eastern Cooperative Oncology Group (ECOG) performance status (PS) 0–3 and sufficient organ function for the chemotherapy. Patients who had received definitive thoracic irradiation; had received antifibrotic agents, such as pirfenidone and nintedanib and had pre-existing histories of AE-ILD were excluded from the study. We enrolled 67 patients in this study and extracted two groups according to a selection flowchart (Fig. 1); that is, patients who received first-line chemotherapy regimens combined with (Bev group) and without (non-Bev group) Bev. Written informed consent for treatment with chemotherapy was obtained from all patients. This study protocol was approved by the institutional review board of Kumamoto University Hospital (approval number: 1448).

### Definition of ILD and AE-ILD

Pre-existing ILD was diagnosed according to clinical features and pretreatment chest high-resolution computed tomography (HRCT) findings. All patients underwent HRCT according to standard clinical practice, and the presence of ILD was evaluated by two pulmonologists (SH and HI). ILD, including idiopathic interstitial pneumonias (IIPs) and connective tissue disease-associated interstitial pneumonia (CTD-IP), was diagnosed when the criteria of ground-glass attenuation, consolidation and reticulation shadow in both lung fields were met. Patients with IIPs, including IPF, were diagnosed according to the international consensus classification of the American Thoracic Society/European Respiratory Society (ATS/ERS) [15]. CTD-IP, including rheumatoid arthritis, was diagnosed based on established criteria [16]. Based on an official ATS/ERS/JRS/ALAT clinical practice guideline [17], CT findings of pre-existing ILD in our study were classified into two groups: usual interstitial pneumonia (UIP) and non-UIP. The UIP pattern is subpleural and basal predominant; the distribution is often heterogeneous, with honeycombing with or without peripheral traction bronchiectasis. The non-UIP pattern included probable UIP pattern, indeterminate for the UIP pattern, and an alternative diagnosis pattern.

Chemotherapy-related AE-ILD was defined as worsening of dyspnoea within 30 days, newly developed bilateral ground-glass abnormality and/or consolidation superimposed on pretreatment interstitial shadows during chemotherapy, no evidence of pulmonary infection and exclusion of alternative causes, including left heart failure, and pulmonary embolism [18].

### Outcomes

The primary endpoint for comparing the Bev and non-Bev groups was the cumulative incidence of AE-ILD in the observation period. Because 46 of the 48 cases received less than or equal to four cycles of first-line chemotherapy, the observation period was defined as the time from the day of initiating first-line chemotherapy (day 1) to day 120 or from day 1 to the day at discontinued chemotherapy due to the following events: cancer progression or death by any cause whichever occurred first or adverse events including AE-ILD. Secondary endpoints were progression-free survival (PFS), calculated as the period from day 1 to the date of disease progression or death by any cause, and overall survival (OS), calculated as the period from day 1 to the date of death by any cause. Patients withdrawn from study without documented progression were censored at the date of last disease assessment. The response to chemotherapy was assessed according to the Response Evaluation Criteria in Solid Tumors version 1.1.

### Data collection and statistical analysis

All clinical and laboratory data were collected from patients’ medical records. Spirometry was performed according to the ATS/ERS consensus guidelines [19]. The JRS reference values of pulmonary function were used to evaluate the percentage of predicted (% predicted) values [20]. Continuous variables are expressed as median (interquartile range [IQR], 25–75%).

This is a retrospective cohort study. To ascertain a normal distribution of variables, Shapiro–Wilk’s test was performed. For univariate analysis, Wilcoxon rank sum tests were used. For categorical variables, Fisher’s exact test was performed. Cumulative incidence analysis (Gray’s test) was performed to verify whether Bev was able to prevent AE-ILD during first-line chemotherapy considering progressive disease (PD) of lung cancer, including cancer-specific death or other events, such as serious adverse events without AE-ILD as competing risks [21]. The log-rank test was used to compare PFD or OS of the two groups in Kaplan–Meier plots. All analyses were performed using R version 3.3.2 (The R Foundation for Statistical Computing, Vienna, Austria), with *p* < 0.05 indicating statistical significance.

## Results

### Patient characteristics

Of 67 patients enrolled in this study, 17 were assigned to the Bev group. Of the remaining 50 patients in the non-Bev group, 19 were excluded because they received different first-line chemotherapy regimens from those in the Bev group, such as docetaxel, nab-paclitaxel (PTX), vinorelbine and S-1. The remaining 31 patients constituted the non-Bev group (Fig. 1).

Baseline characteristics are summarised in Table 1. Median age at the time of first-line chemotherapy was 67.5 years (IQR, 65.0–73.0), and median ECOG PS was 1 (range, 0–3). Six of the 48 patients were never smokers. Stage III and IV diseases were observed in 9 (18.7%) and 39 (81.3%) patients, respectively. The most predominant diagnosis of pre-existing ILD was IIPs (93.7%), and all remaining patients had CTD-IP. With regard to the HRCT findings of ILD, half of the patients had a UIP pattern while the remainder had a non-UIP pattern. The median KL-6 level was elevated, but parameters of respiratory functions, including percent predicted forced vital capacity and percutaneous oxygen saturation (SpO_2_) at rest, were maintained. There were no significant differences between the Bev and non-Bev groups except for the proportion of sex.

**Table 1:** Patient characteristics

	All (*n* = 48)	Bev group (*n* = 17)	Non-Bev group (*n* = 31)	*p* value
Age (years)	67.5 (65.0–73.0)	67.0 (66.0–73.0)	68.0 (66.5–73.0)	0.75
Sex (male, %)	40 (83.3)	17 (100)	23 (74.2)	0.038
Smoking status				0.08
Current or former	42 (87.5)	17 (100)	25 (80.6)	
Never	6 (12.5)	0 (0)	6 (19.4)	
Previous steroid therapy	6 (12.5)	2 (11.8)	4 (12.9)	1.00
ECOG PS				0.37
0–1	40 (83.3)	13 (76.5)	27 (87.1)	
≥2	8 (16.7)	4 (23.5)	4 (12.9)	
Stage				1.00
III	9 (18.7)	3 (17.6)	6 (19.4)	
IV	39 (81.3)	14 (82.4)	25 (80.6)	
Classification of ILD				1.00
IIPs	45 (93.7)	16 (94.1)	29 (93.5)	
Non-IIPs	3 (6.3)	1 (5.9)	2 (6.5)	
ILD pattern				1.00
UIP pattern	24 (50.0)	9 (52.9)	15 (48.4)	
Non-UIP pattern	24 (50.0)	8 (47.1)	16 (51.6)	
SpO_2_ at rest (%)	96.0 (96.0–97.0)	96.0 (95.0–97.0)	96.0 (96.0–97.0)	0.43
FVC (% predicted)	88.5 (79.5–100)	87.5 (78.5–99.5)	90.0 (81.0–100)	0.85
LDH (U/L)	220.0 (194.8–254.0)	228.0 (205.0–253.0)	211.0 (194.5–252.5)	0.48
KL-6 (U/ml)	800.0 (518.5–1190.0)	828.5 (591.0–1311.0)	778.0 (454.0–1120.0)	0.27
CRP (mg/dl)	0.9 (0.3–2.0)	0.9 (0.7–2.3)	0.9 (0.2–1.2)	0.44
First-line regiments				0.96
CDDP + PEM	11 (22.9)	4 (23.5)	7 (22.6)	
CBDCA + PEM	25 (52.1)	10 (58.8)	15 (48.3)	
PEM	4 (8.3)	1 (5.9)	3 (9.7)	
CBDCA + PTX	8 (16.7)	2 (11.8)	6 (19.4)	
PEM-containing regiments	40 (83.3)	15 (88.2)	25 (80.6)	0.69
First-line related AE-ILD	7 (14.5)	0 (0)	7 (22.6)	0.041

Data are expressed as group median (interquartile range) or number of patients (%). The *p* values refer to comparisons between the Bev and non-Bev groups. *ECOG PS* Eastern Cooperative Oncology Group performance status, *ILD* interstitial lung disease, *IIPs* idiopathic interstitial pneumonias, *UIP* usual interstitial pneumonia, *FVC* forced vital capacity, *SpO*_*2*_ percutaneous oxygen saturation, *LDH* lactate dehydrogenase, *KL-6* Krebs von den Lungen-6, *CRP* C-reactive protein, *CDDP* cisplatin, *PEM* pemetrexed, *CBDCA* carboplatin, *PTX* paclitaxel, *AE-ILD* acute exacerbation of interstitial lung disease

### First-line chemotherapy regimens and incidence of AE of pre-existing ILD

**Fig. 2:**
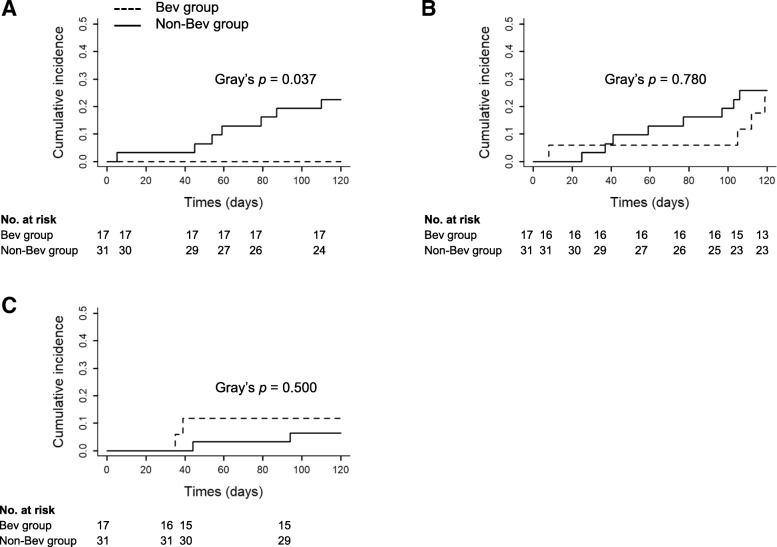
Cumulative incidence curves of three competing risk events during first-line chemotherapy. **a** AE-ILD, (**b**) PD of lung cancer and (**c**) other events without AE-ILD. There was a significant difference in occurrence of AE-ILD between the Bev (dotted line) and non-Bev (solid line) groups (*p* = 0.037, Gray’s test). AE-ILD, acute exacerbation of interstitial lung disease; PD, progressive disease

First-line chemotherapy regimens are shown in Table 1. There were no significant differences in the regimens, excluding Bev treatment, between the two groups (*p* = 0.96). The most common regimen used for first-line chemotherapy was the combination of carboplatin (CBDCA) plus pemetrexed (PEM) in both groups. PEM-containing regimens were administered to 15 (88.2%) and 25 (80.6%) patients in the Bev and non-Bev groups, respectively. The cumulative incidences of AE-ILD at day 120 after first-line chemotherapy were 0% (0 of the 17 patients) and 22.6% (7 of the 31) in the Bev and non-Bev groups, respectively (Tables 1 and 2). In our study, the events of chemotherapy-related AE-ILD are affected by competing risk factors, which are associated with the cessation of chemotherapy as the results of PD of lung cancer or other events, including serious adverse events. Therefore, we defined three competing risk events as follows: the cumulative incidence of chemotherapy-related AE-ILD, PD of lung cancer and other events. Gray’s test [21], which was competing risk analysis during the study period, was performed for both groups. There was a significant difference in AE-ILD between the two groups (Gray’s test, *p* = 0.037; Fig. 2a). However, there were no differences in development of PD of lung cancer and other events between the two groups (Fig. 2b and c). Median time from associated chemotherapy to AE was 61 days. Furthermore, in only patients who received PEM-containing regimens, the incidence of AE-ILD was 0% (0 of the 15) and 24.0% (6 of the 25) in the Bev and non-Bev groups, respectively (Table 2). There also was a significant difference in the incidence of AE-ILD between the two groups (Gray’s test, *p* = 0.044, Additional file 1: Figure S1).

**Table 2:** Frequency of first-line chemotherapeutic regimens and incidence of AE-ILD during first-line chemotherapy

	Bev group (*n* = 17)		Non-Bev group (*n* = 31)	
Regimens	*N*	Incidence of AE	*N*	Incidence of AE
CDDP + PEM	4	0	7	1
CBDCA + PEM	10	0	15	4
PEM	1	0	3	1
CBDCA + PTX	2	0	6	1
Total treatments	17	0	31	7
PEM-containing regimens	15	0	25	6

Data are expressed as number of patients. *AE-ILD* acute exacerbation of interstitial lung disease, *CDDP* cisplatin, *PEM* pemetrexed, *CBDCA* carboplatin, *PTX* paclitaxel

### Risk factors of chemotherapy-related AE of ILD

Next, we compared clinical parameters between 7 patients with and 41 without AE-ILD during first-line chemotherapy to evaluate risk factors of AE-ILD. Patient age (*p* = 0.019) and administration of Bev (*p* = 0.041) were significant (Table 3). Although we would like to verify hazard ratios of Bev for AE-ILD, the administration of Bev inhibited AE-ILD completely during first-line chemotherapy, so that the hazard ratio was not obtained.

**Table 3:** Comparison of the clinical factors between patients with and without AE-ILD

	AE-ILD (−) (*n* = 41)	AE-ILD (+) (*n* = 7)	*p* value
Age (years)	69.0 (66.0–75.0)	65.0 (63.0–66.5)	0.019
Sex (male, %)	34 (82.9)	6 (85.7)	1.00
Smoking status			0.57
Current or former	35 (85.4)	7 (100)	
Never	6 (14.6)	0 (0)	
Previous steroid therapy	4 (9.8)	2 (28.6)	0.21
ECOG PS			0.59
0–1	33 (80.5)	7 (100)	
≥2	8 (19.5)	0 (0)	
Stage			1.00
III	8 (19.5)	1 (14.3)	
IV	33 (80.5)	6 (85.7)	
Classification of ILD			1.00
IIPs	38 (92.7)	7 (100)	
Non-IIPs	3 (7.3)	0 (0)	
ILD pattern			0.42
UIP pattern	22 (53.7)	2 (28.6)	
Non-UIP pattern	19 (46.3)	5 (71.4)	
SpO_2_ at rest (%)	96.0 (95.0–9.07)	96.0 (95.0–97.0)	0.82
FVC (% predicted)	89.0 (78.5–100)	82.0 (81.0–101.0)	0.74
LDH (U/L)	219.0 (194.0–253.0)	224.0 (202.5–257.5)	0.74
KL-6 (U/ml)	789.0 (530.2–1196.0)	893.0 (537.0–1048.0)	0.86
CRP (mg/dl)	0.9 (0.3–1.5)	0.9 (0.8–2.8)	0.58
First-line regimens			0.86
CDDP + PEM	10 (24.4)	1 (14.3)	
CBDC + PEM	21 (51.2)	4 (57.1)	
PEM	3 (7.3)	1 (14.3)	
CBDCA + PTX	7 (17.1)	1 (14.3)	
Bev-containing regimens	17 (41.5)	0 (0)	0.041
PEM-containing regimens	34 (82.9)	6 (85.7)	1.00

Data are expressed as group median (interquartile range) or number of patients (%). The *p* values refer to comparisons between patients with and without AE-ILD. *ECOG PS* Eastern Cooperative Oncology Group performance status, *ILD* interstitial lung disease, *IIPs* idiopathic interstitial pneumonias, *UIP* usual interstitial pneumonia, *FVC* forced vital capacity, *SpO*_*2*_ percutaneous oxygen saturation, *LDH* lactate dehydrogenase, *KL-*6 Krebs von den Lungen-6, *CRP* C-reactive protein, *CDDP* cisplatin, *PEM* pemetrexed, *CBDCA* carboplatin, *PTX* paclitaxel

We also examined the cumulative incidence of AE-ILD during subsequent chemotherapy beyond first-line chemotherapy. In the Bev group, 15 of the 17 patients (88.2%) received subsequent chemotherapy, and 2 (13.3%) had AE-ILD, compared with 24 of 31 (77.4%) and 5 (20.8%) patients, respectively, in the non-Bev group (Table 4). Consequently, the overall-cumulative incidence of AE-ILD in all of our patients was 29.2% (14 of the 48). All patients who had AE-ILD were treated with high-dose corticosteroids, including methylprednisolone pulse therapy; however, 9 of 14 died of respiratory failure associated with AE (Table 4).

**Table 4:** Outcomes of 14 patients with AE-ILD after chemotherapy

	No.	Treatment line	Associated regimens	Onset of AE (days from chemotherapy)	Outcome	Survival time (days from onset of AE)
Non-Bev group	1	First	CDDP + PEM	79	Recover	125
	2	First	CBDCA + PEM	110	Dead	87
	3	First	CBDCA + PEM	5	Recovered	125
	4	First	CBDCA + PEM	59	Dead	77
	5	First	CBDCA + PEM	45	Dead	11
	6	First	CBDCA + PEM	54	Recovered	626
	7	First	CBDCA + PTX	87	Dead	36
	8	Second	DOC	169	Recovered	276
	9	Second	DOC	193	Recovered	522
	10	Second	S-1	120	Dead	5
	11	Second	DOC	426	Dead	4
	12	Third	DOC	372	Dead	67
Bev group	13	Second	nab-PTX	156	Dead	34
	14	Third	DOC	386	Dead	17

*AE* acute exacerbation, *Bev* bevacizumab, *CDDP* cisplatin, *PEM* pemetrexed, *CBDCA* carboplatin, *PTX* paclitaxel, *DOC* docetaxel, *nab-PTX* nab-paclitaxel, *VNR* vinorelbine

### Comparison of clinical outcomes

**Fig. 3:**
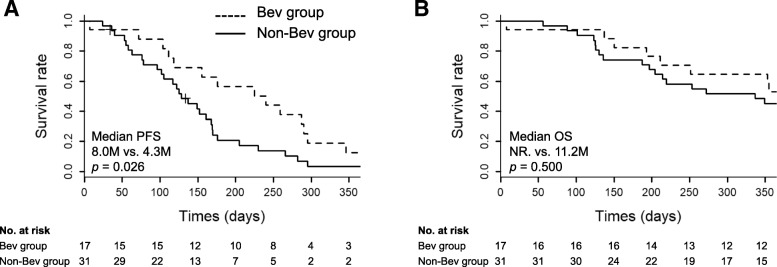
Kaplan–Meier comparisons in the Bev and non-Bev groups. **a** Progression-free survival (PFS). **b** Overall survival (OS). Crosses denote censoring events. There was a significant difference in median PFS between the Bev (dotted line) and non-Bev (solid line) groups (*p* = 0.026). NR., not reached

Kaplan–Meier comparisons of survival curves in the Bev and non-Bev groups are shown in Fig. 3. Median PFS was significantly longer in the Bev than in the non-Bev groups (8.0 months; 95% confidence interval [CI], 3.7–9.7 vs. 4.3 months; 95% CI, 3.2–5.6; *p* = 0.026; Fig. 3a). Median OS was not significantly different between the groups (not reached; 95% CI, 6.4–not reached in the Bev group vs. 11.2 months; 95% CI, 6.6–not reached in the non-Bev group; *p* = 0.500; Fig. 3b).

## Discussion

To our knowledge, this is the first study to show the inhibitory effect of Bev, an antibody against VEGF, on chemotherapy-related AE-ILD in patients with lung cancer. The cumulative incidence of AE-ILD was significantly lower in the Bev than in the non-Bev groups (*p* = 0.037). Only in cases receiving PEM-containing regimens, there was also a significant difference between the two groups (*p* = 0.044). Our study suggested that VEGF inhibition might suppress the risk of chemotherapy-related AE-ILD. Further, the administration of Bev in first-line chemotherapy was associated with a significantly longer median PFS (*p* = 0.026). Therefore, first-line chemotherapy regimens containing Bev may be safe and favourable for patients with non-squamous NSCLC with pre-existing ILD.

The mechanism of the inhibitory effect of Bev on chemotherapy-related AE-ILD remains unclear. VEGF, a potent stimulator of angiogenesis and vascular permeability, has an important role in lung diseases, including fibrosis, injury and cancer [8, 22]. In patients with ARDS, increasing levels of plasma VEGF are associated with a worse prognosis [10]. Treatment of VEGF receptor inhibition, such as with nintedanib, is beneficial for patients with IPF [23], and adsorptive removal of circulating VEGF by polymyxin B haemoperfusion improves pulmonary oxygenation in AE-IPF [24]. In contrast, significant amounts of VEGF exist in the normal lung and are involved in lung maintenance and repair after injury [9]. Downregulation of VEGF synthesis has been observed in the lung of patients with ARDS [12]. Taken together, the acts of VEGF are considered to have pleiotropic properties. There also are conflicting reports by assessing the biological effect of VEGF in experimental animal models. The inhibition of VEGF attenuated bleomycin-induced lung fibrosis [25, 26], whereas Murray et al. [27] reported the opposite results, that is, that transgenic overexpression of VEGF attenuated the lung injury and fibrosis. Recently, an interesting study by Barratt et al. [14], in which differential expression of VEGF-A isoforms is critical in the developing pulmonary fibrosis, has been reported. It generally is known that Bev targets all isoforms of VEGF-A. The precise role of VEGF in lung diseases, whether either protective or detrimental, still is being debated. Therefore, to elucidate the association of chemotherapy-related AE-ILD and VEGF and the influence of administration of Bev in the coexistence of lung cancer and ILD, further studies are required. Interestingly, a prospective, randomized controlled study is currently ongoing in Japan for patients with NSCLC and IPF to evaluate the efficacy and safety of chemotherapy with or without nintedanib, which inhibits receptor tyrosine kinase signaling by VEGF, platelet-derived growth factor, and fibroblast growth factors [28]. The goal of the study is to demonstrate whether the nintedanib combined chemotherapeutic regimens prolong the interval to AE-IPF, and the results of this study are expected in the future.

In clinical practice, patients with lung cancer with ILD have been treated carefully with cytotoxic chemotherapy agents, because the pre-existing ILDs have proved to be significant risk factors for chemotherapy-related AE, resulting in poor survival [1–3]. It has been known that the incidence of AE-ILD during the whole chemotherapy process was higher than during its natural course [29]. To date, one prospective study and a few retrospective studies have demonstrated the validity of chemotherapy for patients with NSCLC with ILD, allowing for the risk of AE associated with chemotherapy [4, 5, 29]. Minegishi et al. [4] reported that the combination of CBDCA and PTX was effective and safe for patients with NSCLC with IIPs in the prospective study. Similarly, Chen et al. [29] also showed, by conducting systematic reviews and meta-analyses in NSCLC with ILD, that the regimen of CBDCA and PTX would not be related to higher incidence of AE-ILD and could be used safely, even though the limited number of studies and patients were available. In our study, 1 of the 6 patients received CBDCA plus PTX without Bev and suffered AE. However, there is still no widely accepted consensus on supporting an optimal treatment strategy for the comorbidity of lung cancer and ILD.

In our study, the combinations of CBDCA and PEM with or without Bev were used most frequently as first-line chemotherapy for NSCLC with ILD. AE-ILD related to PEM-containing regimens without Bev as first-line chemotherapy was observed in 24.0% (6 of the 25 patients), which was greater compared with the 5.5–12% rates in previous studies and postmarketing surveillance in Japan [30–32]. The reason for a higher incidence of AE-ILD in our study is unclear, but this difference may be attributed to small sample size, including patients with ECOG PS 2–3, limited patients with non-squamous NSCLC and the variation in therapeutic regimens. Although the interpretation of the results from our study is limited, even with the increased risk of AE, the complication was significantly suppressed by the addition of Bev. The significance of this suppression should be evaluated in the future.

We also evaluated the efficacy of Bev as the anticancer drug. Several studies have shown that PFS is significantly longer when Bev is administered to patients with advanced NSCLC without ILD [33, 34]. Two reports were intended for patients with lung cancer with pre-existing ILD receiving Bev [35, 36]. Shimizu et al. [35] examined the safety and efficacy of CBDCA plus PTX with or without Bev in patients with non-squamous NSCLC and ILD. They reported that PFS and OS tended to be longer, but not significantly, in the Bev than in non-Bev groups (PFS, 5.5 vs. 4.4 months; OS, 16.1 vs. 9.7 months), although there was no significant difference in the incidence of AE-ILD between CBDCA plus PTX with and without Bev [35]. Another study showed that PFS and OS of patients treated with CBDCA plus PTX with Bev were 7.2 and 8.5 months, respectively, resulting in a 12% incidence rate of AE-ILD [36]. Our result showed that the Bev group, compared with the non-Bev group, was associated with a longer median PFS (8.0 vs. 4.3 months, *p* = 0.026), which was similar to results of the aforementioned reports. However, there are limitations in the interpretation of these results because all of the above studies including ours were not randomised, and the combination of anticancer agents varied. Further studies including randomised controlled trials are necessary to evaluate the efficacy of Bev-containing regimens and the optimal combination chemotherapy regimen to Bev in patients with lung cancer with ILD.

This study has several limitations. First, this was a small, retrospective and not a randomised controlled study, giving rise to selection bias. Second, diagnosis of ILD was based on chest CT findings and not on histological findings. Third, this study included only Japanese patients. Some studies suggest racial differences in susceptibility to chemotherapy-induced AE-ILD [3, 37]. Further studies are needed to validate whether our results could apply equally to other racial groups. Finally, each attending physician from our institutes usually selects first-line chemotherapeutic regimens based on NCCN Clinical Practice Guidelines [38]. The addition of Bev to platinum based chemotherapy is recommended, if possible. However, there is insufficient evidence and no criteria to determine its use in patients with non-squamous NSCLC who have ILD. Therefore, the decision regarding the choice of Bev was made by the attending physicians in each case.

## Conclusions

This preliminary study suggests that the inhibition of VEGF with Bev might reduce the incidence of chemotherapy-related AE-ILD, and first-line chemotherapeutic regimens containing Bev could be optimal in patients with advanced non-squamous NSCLC with pre-existing ILD. Further large-scale, randomised, controlled studies are needed to confirm the inhibitory effect of Bev on chemotherapy-related AE-ILD and to develop better therapeutic managements for patients with lung cancer and pre-existing ILD.

## Additional file

Additional file 1: **Figure S1.** Cumulative incidence curves of AE-ILD in only patients who received PEM-containing regimens during first-line chemotherapy. (PPTX 381 kb)

## Abbreviations

AE: Acute exacerbation
ARDS: Acute respiratory distress syndrome
ATS: American Thoracic Society
Bev: Bevacizumab
CBDCA: Carboplatin
CDDP: Cisplatin
CRP: C-reactive protein
CTD-IP: Connective tissue disease-associated interstitial pneumonia
DOC: Docetaxel
ECOG: Eastern Cooperative Oncology Group
ERS: European Respiratory Society
FVC: Forced vital capacity
HRCT: High-resolution computed tomography
IIPs: Idiopathic interstitial pneumonias
ILD: Interstitial lung disease
IPF: Idiopathic pulmonary fibrosis
JRS: Japanese Respiratory Society
KL-6: Krebs von den Lungen-6
LDH: Lactate dehydrogenase
NCCN: National Comprehensive Cancer Network
NSCLC: Non-small cell lung cancer
OS: Overall survival
PD: Progressive disease
PEM: Pemetrexed
PFS: Progression-free survival
PS: Performance status
SpO_2_: Percutaneous oxygen saturation
UIP: Usual interstitial pneumonia
VEGF: Vascular endothelial growth factor
VNR: Vinorelbine

## References

[1] Raghu G, Nyberg F, Morgan G (2004). The epidemiology of interstitial lung disease and its association with lung cancer. Br J Cancer.

[2] Archontogeorgis K, Steiropoulos P, Tzouvelekis A, Nena E, Bouros D. Lung cancer and interstitial lung diseases: a systematic review. Pulm Med. 2012:315918. 10.1155/2012/315918 PMC3414065 22900168

[3] Kudoh S, Kato H, Nishiwaki Y, Fukuoka M, Nakata K, Ichinose Y (2008). Interstitial lung disease in Japanese patients with lung cancer: a cohort and nested case-control study. Am J Respir Crit Care Med.

[4] Minegishi Y, Sudoh J, Kuribayasi H, Mizutani H, Seike M, Azuma A (2011). The safety and efficacy of weekly paclitaxel in combination with carboplatin for advanced non-small cell lung cancer with idiopathic interstitial pneumonias. Lung Cancer.

[5] Watanabe N, Taniguchi H, Kondoh Y, Kimura T, Kataoka K, Nishiyama O (2013). Efficacy of chemotherapy for advanced non-small cell lung cancer with idiopathic pulmonary fibrosis. Respiration..

[6] Antoniou KM, Wells AU (2013). Acute exacerbations of idiopathic pulmonary fibrosis. Respiration..

[7] McKeown S, Richter AG, O'Kane C, McAuley DF, Thickett DR (2009). MMP expression and abnormal lung permeability are important determinants of outcome in IPF. Eur Respir J.

[8] Weis SM, Cheresh DA (2005). Pathophysiological consequences of VEGF-induced vascular permeability. Nature..

[9] Barratt S, Medford AR, Millar AB (2014). Vascular endothelial growth factor in acute lung injury and acute respiratory distress syndrome. Respiration..

[10] Thickett DR, Armstrong L, Christie SJ, Millar AB (2001). Vascular endothelial growth factor may contribute to increased vascular permeability in acute respiratory distress syndrome. Am J Respir Crit Care Med.

[11] Kulkarni YM, Dutta S, Iyer AK, Venkatadri R, Kaushik V, Ramesh V (2016). A proteomics approach to identifying key protein targets involved in VEGF inhibitor mediated attenuation of bleomycin-induced pulmonary fibrosis. Proteomics..

[12] Maitre B, Boussat S, Jean D, Gouge M, Brochard L, Housset B (2001). Vascular endothelial growth factor synthesis in the acute phase of experimental and clinical lung injury. Eur Respir J.

[13] Mura M, Han B, Andrade CF, Seth R, Hwang D, Waddell TK (2006). The early responses of VEGF and its receptors during acute lung injury: implication of VEGF in alveolar epithelial cell survival. Crit Care.

[14] Barratt SL, Blythe T, Jarrett C, Ourradi K, Shelley-Fraser G, Day MJ (2017). Differential expression of VEGF-Axxx isoforms is critical for development of pulmonary fibrosis. Am J Respir Crit Care Med.

[15] Travis WD, Costabel U, Hansell DM, King TE, Lynch DA, Nicholson AG (2013). ATS/ERS Committee on idiopathic interstitial pneumonias. An official American Thoracic Society/European Respiratory Society statement: update of the international multidisciplinary classification of the idiopathic interstitial pneumonias. Am J Respir Crit Care Med.

[16] Vij R, Strek ME (2013). Diagnosis and treatment of connective tissue disease-associated interstitial lung disease. Chest..

[17] Raghu G, Remy-Jardin M, Myers JL, Richeldi L, Ryerson CJ, Lederer DJ (2018). Diagnosis of idiopathic pulmonary fibrosis. An official ATS/ERS/JRS/ALAT clinical practice guideline. Am J Respir Crit Care Med.

[18] Collard HR, Moore BB, Flaherty KR, Brown KK, Kaner RJ, King TE (2007). Idiopathic pulmonary fibrosis clinical research network investigators. Acute exacerbations of idiopathic pulmonary fibrosis. Am J Respir Crit Care Med.

[19] Wanger J, Clausen JL, Coates A, Pedersen OF, Brusasco V, Burgos F (2005). Standardisation of the measurement of lung volumes. Eur Respir J.

[20] Kubota M, Kobayashi H, Quanjer PH, Omori H, Tatsumi K, Kanazawa M (2014). Clinical pulmonary functions Committee of the Japanese Respiratory Society. Reference values for spirometry, including vital capacity, in Japanese adults calculated with the LMS method and compared with previous values. Respir Investig.

[21] Gray RJ (1988). A class of K-sample tests for comparing the cumulative incidence of a competing risk. Ann Stat.

[22] Simler NR, Brenchley PE, Horrocks AW, Greaves SM, Hasleton PS, Egan JJ (2004). Angiogenic cytokines in patients with idiopathic interstitial pneumonia. Thorax..

[23] Richeldi L, du Bois RM, Raghu G, Azuma A, Brown KK, Costabel U (2014). INPULSIS trial investigators. Efficacy and safety of nintedanib in idiopathic pulmonary fibrosis. N Engl J Med.

[24] Oishi K, Mimura-Kimura Y, Miyasho T, Aoe K, Ogata Y, Katayama H (2013). Association between cytokine removal by polymyxin B hemoperfusion and improved pulmonary oxygenation in patients with acute exacerbation of idiopathic pulmonary fibrosis. Cytokine..

[25] Hamada N, Kuwano K, Yamada M, Hagimoto N, Hiasa K, Egashira K (2005). Anti-vascular endothelial growth factor gene therapy attenuates lung injury and fibrosis in mice. J Immunol.

[26] Ou XM, Li WC, Liu DS, Li YP, Wen FQ, Feng YL (2009). VEGFR-2 antagonist SU5416 attenuates bleomycin-induced pulmonary fibrosis in mice. Int Immunopharmacol.

[27] Murray LA, Habiel DM, Hohmann M, Camelo A, Shang H, Zhou Y (2017). Antifibrotic role of vascular endothelial growth factor in pulmonary fibrosis. JCI Insight.

[28] Otsubo K, Kishimoto J, Kenmotsu H, Minegishi Y, Ichihara E, Shiraki A (2018). Treatment rationale and design for J-SONIC: a randomized study of carboplatin plus nab-paclitaxel with or without nintedanib for advanced non-small-cell lung cancer with idiopathic pulmonary fibrosis. Clin Lung Cancer.

[29] Chen YJ, Chen LX, Han MX, Zhang TS, Zhou ZR, Zhong DS (2015). The efficacy and safety of chemotherapy in patients with nonsmall cell lung cancer and interstitial lung disease: a PRISMA-compliant bayesian meta-analysis and systematic review. Medicine (Baltimore).

[30] Choi MK, Hong JY, Chang W, Kim M, Kim S, Jung HA (2014). Safety and efficacy of gemcitabine or pemetrexed in combination with a platinum in patients with non-small-cell lung cancer and prior interstitial lung disease. Cancer Chemother Pharmacol.

[31] Kato M, Shukuya T, Takahashi F, Mori K, Suina K, Asao T (2014). Pemetrexed for advanced non-small cell lung cancer patients with interstitial lung disease. BMC Cancer.

[32] Tomii K, Kato T, Takahashi M, Noma S, Kobashi Y, Enatsu S (2017). Pemetrexed-related interstitial lung disease reported from post marketing surveillance. Jpn J Clin Oncol.

[33] Sandler A, Gray R, Perry MC, Brahmer J, Schiller JH, Dowlati A (2006). Paclitaxel-carboplatin alone or with bevacizumab for non-small-cell lung cancer. N Engl J Med.

[34] Niho S, Kunitoh H, Nokihara H, Horai T, Ichinose Y, Hida T (2012). JO19907 study group. Randomized phase II study of first-line carboplatin-paclitaxel with or without bevacizumab in Japanese patients with advanced non-squamous non-small-cell lung cancer. Lung Cancer.

[35] Shimizu R, Fujimoto D, Kato R, Otoshi T, Kawamura T, Tamai K (2014). The safety and efficacy of paclitaxel and carboplatin with or without bevacizumab for treating patients with advanced nonsquamous non-small cell lung cancer with interstitial lung disease. Cancer Chemother Pharmacol.

[36] Enomoto Y, Kenmotsu H, Watanabe N, Baba T, Murakami H, Yoh K (2015). Efficacy and safety of combined carboplatin, paclitaxel, and bevacizumab for patients with advanced non-squamous non-small cell lung cancer with pre-existing interstitial lung disease: a retrospective multi-institutional study. Anticancer Res.

[37] Azuma A, Hagiwara K, Kudoh S (2008). Basis of acute exacerbation of idiopathic pulmonary fibrosis in Japanese patients. Am J Respir Crit Care Med.

[38] Ettinger DS, Wood DE, Akerley W, Bazhenova LA, Borghaei H, Camidge DR (2014). Non-small cell lung cancer, version 1.2015. J Natl Compr Cancer Netw.

