# Supplementary material for: Protective effect of bevacizumab on chemotherapy-related acute exacerbation of interstitial lung disease in patients with advanced non-squamous non-small cell lung cancer
Source: BMC Pulm Med. 2019 Apr 2;19:72. doi: 10.1186/s12890-019-0838-2 (PMC6446385; doi:10.1186/s12890-019-0838-2)
Supplement: Supplementary file 1 — Figure S1. Cumulative incidence curves of AE-ILD in only patients who received PEM-containing regimens during first-line chemotherapy. (PPTX 381 kb) [file 12890_2019_838_MOESM1_ESM.pptx]

## Slide 1
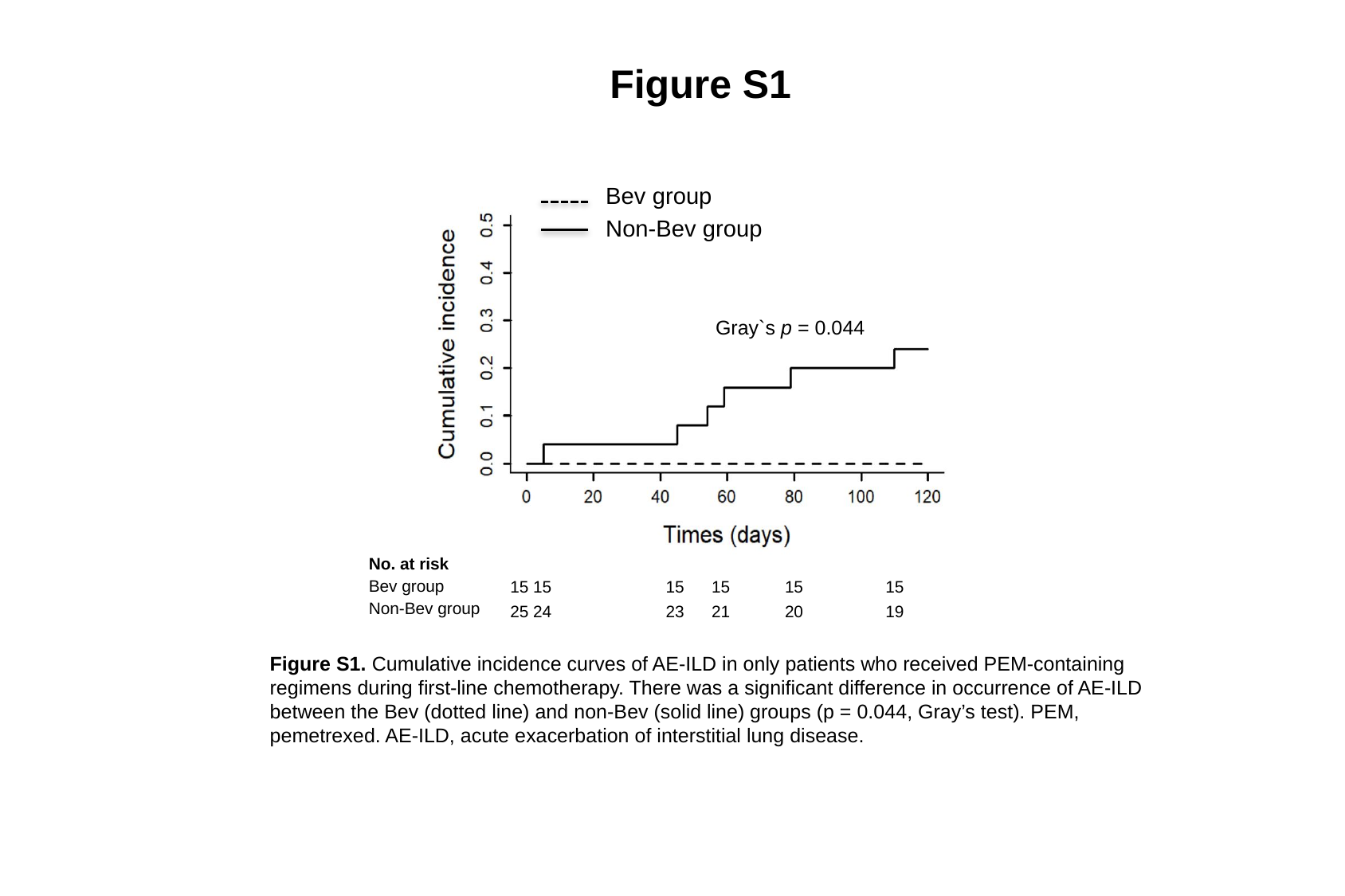

Figure S1
Bev group
Non-Bev group
Gray`s p = 0.044
No. at risk
Bev group
15 15 15 15 15 15
Non-Bev group
25 24 23 21 20 19
Figure S1. Cumulative incidence curves of AE-ILD in only patients who received PEM-containing regimens during first-line chemotherapy. There was a significant difference in occurrence of AE-ILD between the Bev (dotted line) and non-Bev (solid line) groups (p = 0.044, Gray’s test). PEM, pemetrexed. AE-ILD, acute exacerbation of interstitial lung disease.
